# Supplementary material for: Enteroaggregative Escherichia coli is associated with antibiotic resistance and urinary tract infection symptomatology
Source: PeerJ. 2021 Aug 24;9:e11726. doi: 10.7717/peerj.11726 (PMC8395569; doi:10.7717/peerj.11726)
Supplement: Supplemental Information 1 [file peerj-09-11726-s001.docx]

**Table 1S. Genotypes of the isolates analyzed in this work**.

| **ID** | ***aatA*** | ***aggR*** | ***aapA*** |
| --- | --- | --- | --- |
| 2113 | - | - | - |
| 2123 | - | - | - |
| 2176 | - | - | - |
| 2194 | - | - | - |
| 2198 | - | - | - |
| 2210 | - | - | - |
| 2213 | - | - | - |
| 2249 | + | - | - |
| 2254 | - | - | - |
| 2296 | - | + | + |
| 2302 | + | - | + |
| 2368 | + | - | - |
| 2379 | - | - | - |
| 2390 | - | - | - |
| 2396 | - | - | - |
| 2477 | - | - | - |
| 2486 | - | - | - |
| 2490 | + | + | + |
| 2495 | - | - | - |
| 2578 | + | + | + |
| 3136 | - | + | - |
| 3215 | - | - | - |
| 3238 | + | - | + |
| 3332 | + | + | - |
| 4011 | - | - | - |
| 4012 | - | - | - |
| 4013 | - | - | - |
| 4014 | - | - | + |
| 4015 | - | - | + |
| 4016 | - | - | - |
| 4017 | - | - | - |
| 4018 | - | - | + |
| 4019 | + | + | + |
| 4020 | - | - | - |
| 4021 | - | + | + |
| 4022 | - | - | - |
| 4023 | - | - | - |
| 4024 | - | - | - |
| 4025 | - | - | - |
| 4026 | - | - | - |
| 4027 | - | - | - |
| 4028 | - | - | - |
| 4029 | - | + | + |
| 4030 | - | - | - |
| 4031 | - | - | - |
| 4032 | - | - | - |
| 4033 | - | - | - |
| 4034 | - | + | + |
| 4035 | - | + | - |
| 4036 | - | - | - |
| 4037 | - | + | + |
| 4038 | - | - | + |
| 4039 | - | - | + |
| 4040 | - | + | - |
| 4041 | - | + | + |
| 4042 | - | - | - |
| 4043 | - | + | + |
| 4044 | - | - | - |
| 4045 | - | - | - |
| 4046 | - | - | - |
| 4048 | + | + | + |
| 4049 | - | + | + |
| 4050 | + | + | - |
| 4051 | - | - | - |
| 4052 | - | - | - |
| 4054 | - | - | - |
| 4055 | - | - | + |
| 4056 | + | - | + |
| 4057 | - | + | + |
| 4058 | - | + | + |
| 4059 | + | - | - |
| 4060 | - | - | - |
| 4061 | - | - | - |
| 4062 | - | - | - |
| 4063 | - | - | - |
| 4064 | - | - | - |
| 4065 | - | - | - |
| 4066 | - | + | - |
| 4067 | - | - | - |
| 4068 | + | - | - |
| 4069 | - | - | - |
| 4070 | + | + | - |
| 4071 | - | - | - |
| 4072 | - | - | - |
| 4073 | - | - | - |
| 4074 | - | - | - |
| 4075 | - | - | - |
| 4076 | - | - | - |
| 4077 | - | - | - |
| 4078 | - | - | - |
| 4079 | - | - | - |
| 4080 | - | - | - |
| 4081 | - | - | - |
| 4082 | - | - | - |
| 4083 | - | - | - |
| 4084 | - | - | - |
| 4085 | - | - | - |
| 4086 | - | + | - |
| 4087 | - | - | - |
